# Current cough and sputum assessed by the cough and sputum assessment-questionnaire (CASA-Q) is associated with quality of life impairment in cystic fibrosis

**DOI:** 10.1186/s12890-023-02701-3

**Published:** 2023-11-21

**Authors:** Lucie Charon, Claire Launois, Jeanne-Marie Perotin, Bruno Ravoninjatovo, Pauline Mulette, Julien Ancel, Thomas Guillard, Anaëlle Muggeo, Valérian Dormoy, Muriel Griffon, Sophie Carré, François Lebargy, Gaëtan Deslée, Sandra Dury

**Affiliations:** 1 Service des Maladies Respiratoires et Allergiques, CHU de Reims, Reims, France; 2 Service des Maladies Respiratoires et Allergiques, Université de Reims Champagne-Ardenne, INSERM, CHU de Reims, P3Cell, U 1250, Reims, France; 3 Laboratoire de Bactériologie-Virologie-Hygiène hospitalière- Parasitologie-Mycologie, Université de Reims Champagne-Ardenne, INSERM, CHU de Reims, P3Cell, Reims, U 1250 France; 4 INSERM UMRS 1250, Université de Reims Champagne-Ardenne, Reims, France; 5 Service des Maladies Respiratoires et Allergiques, Université de Reims Champagne-Ardenne, EA7509 IRMAIC, CHU de Reims, Reims, France; 6 EA7509 IRMAIC, Université de Reims Champagne-Ardenne, Reims, France

**Keywords:** Cystic fibrosis, Quality of life, CASA-Q, CFQ-R, Cough, Sputum

## Abstract

**Background:**

Cough and sputum are major symptoms in cystic fibrosis (CF) that contribute to the impairment of quality of life.

**Methods:**

This prospective single centre cross-sectional pilot study aimed to evaluate the results of a self-administered questionnaire assessing cough and sputum symptoms (2 domains), and their impact (2 domains) on daily activities in the previous week, named the Cough and Sputum Assessment Questionnaire (CASA-Q) in CF adult patients at stable state, and to analyse associations with clinical, functional, microbiological, radiological data, and two quality of life scales: the Cystic Fibrosis Questionnaire Revised (CFQ-R) and the Saint George Respiratory Questionnaire (SGRQ).

**Results:**

Forty-eight patients were included in this analysis (69% men; median age of 27.8 ± 8.1 years; median body mass index of 21.8 + 3.3 kg/m²; mean FEV_1_ of 64 ± 30% of the predicted value). The mean values of the CASA-Q domains were 58 ± 23 for cough symptoms, 77 ± 24 for cough impact, 62 ± 25 for sputum symptoms and 84 ± 21 for sputum impact. Impairment in CASA-Q cough and sputum domains was associated with dyspnea mMRC scale (p < 0.005 for all 4 domains of CASA-Q) and exacerbations in the previous year (p < 0.05 for CASA-Q symptoms domains). We also found correlations between all domains of the CASA-Q and quality of life questionnaires including SGRQ (p < 0.001) and to a lesser extend CFQ-R. We identified a clinical phenotype (female gender, ΔF508 heterozygous mutation, dyspnea mMRC scale) associated with an impairment of CASA-Q score and quality of life using a 2-step cluster analysis.

**Conclusions:**

CASA-Q allows the assessment of cough and sputum in CF adult patients and is associated with quality of life impairment. This simple easy-to-use tool could be used in routine clinical practice and in clinical studies to assess cough and sputum in CF patients.

**Trial Registration:**

The study was registered on ClinicalTrials.gov (NCT02924818, first posted on 5th October 2016).

**Supplementary Information:**

The online version contains supplementary material available at 10.1186/s12890-023-02701-3.

## Background

Cystic fibrosis (CF) is the most common autosomal recessive life-limiting disease worldwide and currently affects more than 70 000 patients mainly those of European descent [1]. Over 2000 mutations are known to affect the CFTR gene leading to progressive mucociliary clearance defect and bronchiectasis. It may be responsible for chronic cough, airway obstruction by sputum, and recurrent pulmonary infections highly impairing quality of life [2] [3].

Several Patient Reported Outcomes (PRO) questionnaires measure disease impact on health- related quality of life, including the Cystic Fibrosis Questionnaire Revised (CFQ-R) [4] and the Saint George Respiratory Questionnaire (SGRQ) [5]. These questionnaires include cough and sputum symptoms but do not offer in-depth questioning of these symptoms. Moreover, the SGRQ is respiratory specific but not CF specific.

The Cough and Sputum Assessment Questionnaire (CASA-Q), developed in 2008 for chronic obstructive pulmonary disease (COPD) patients, is correlated in COPD with quality of life as measured by the Saint George Respiratory Questionnaire [6]. Since then, it has been used in other respiratory chronic diseases such as asthma [7], idiopathic pulmonary fibrosis [8], and tuberculosis [9]. Recently the CASA-Q was used to compare two techniques of daily airway clearance in CF patients [10].

Our objectives were to describe the results of the CASA-Q in CF adult patients, to analyse the associations between CASA-Q and clinical, functional, microbiological, and radiological data, and finally to study the correlation between CASA-Q and quality of life assessed by the CFQ-R and SGRQ.

## Methods

### Study protocol

The patients were prospectively recruited from the Department of Respiratory Diseases - Reims University Hospital, France between November 2016 and December 2019 and included in the RINNOPARI cross-sectional study (Recherche et INNOvation en PAthologie Respiratoire Inflammatoire), an observational cohort of inflammatory chronic lung diseases (NCT02924818, first posted on 5th October 2016). Each patient signed a written informed consent form.

Consecutive patients followed up for CF in the Department of Respiratory Diseases at Reims University Hospital were considered for inclusion if they were at least 18 years old. Exclusion criteria were previous or planned lung transplantation and patients requiring an urgent visit or any ongoing or recent medical condition in the last 4 weeks, including pulmonary exacerbations. We recorded and registered demography, clinical characteristics, pulmonary function test results, exercise tolerance assessed by the 6-minute walking test results (6MWT), sputum microbiological data and thoracic computerized tomography (CT) scan features on an electronic case report form.

For microbiological analysis, sputum samples at inclusion were liquefied by N-acetylcysteine and 1/1000 dilutions were cultured in Columbia blood and chocolate agar (Thermo Fisher Scientific) at 37 °C for 48 h. Colonies were identified by MALDI-TOF mass spectrometry (MALDI Biotyper®, Bruker Daltonics). Chronic infection by *Pseudomonas aeruginosa* was defined according to Leeds criteria [11]. A similar definition was used to record chronic infections by *Staphylococcus aureus*, *Stenotrophomonas maltophilia, Burkolderia cepacia* and *Achromobacter xylosoxidans*.

Thoracic CT scans performed within 6 months either before or after the inclusion in the study were analyzed by two independent investigators (SD, GD) with a final consensus interpretation. All CT scans were performed with the patient in the supine position at end-inspiratory volume using multidetector CT scanners. One- to 5- mm-thick slices at 5- to 10-mm intervals were analyzed from the lung apices to the lung bases. The diagnosis of bronchiectasis was defined according to the Fleischner Society as dilated bronchial lumen relative to the adjacent pulmonary artery, absence of bronchial tapering, and visualization of bronchi within 1 cm of the pleural surface [12]. The extent of bronchiectasis (0–3), the thickness of the bronchial wall (0–3) and small airways abnormalities (0–1), were quantified for each lobe (lingula was considered as a separated lobe) and a total score (sum of the score of each lobe) was calculated according to Ooi et al. score. The total score ranked from 0 to 42, a higher score suggesting a higher impairment [13].

### Cough and sputum assessment questionnaire (CASA-Q)

The CASA-Q is a self-administered questionnaire assessing cough and sputum symptoms, and their impact on daily activities over the 7 previous days [6]. The score contains four domains: cough symptoms (3 items), cough impact (8 items), sputum symptoms (3 items), and sputum impact (6 items). Each item is answered on a scale ranging from “never” to “always” for frequency and from “not at all” to “a lot/extremely” for intensity. For each domain, items were summed and rescaled to obtain a score ranging from 0 to 100, with a higher score reflecting milder respiratory impairment. As previously described, the data were analysed for each domain separately and no total score (sum of the scores of the four CASA-Q domains) was calculated [6, 14].

### Quality of life scales

The health-related quality of life of CF patients was assessed using two questionnaires: (1) the CFQ-R, a CF-specific questionnaire that includes 50 items and provides insight on respiratory and digestive functions and overall welfare during the last 2 weeks. Each score is standardized from 0 to 100, a higher score reflecting a better quality of life [15]; (2) the SGRQ which measures the impact of lung disease on quality of life and well-being and consists of 50 questions assessing symptoms, and their impact on activity and quality of life over the last 4 weeks. The total score ranges from 0 to 100, a higher score indicating a worse quality of life [5].

### Primary and secondary endpoints

The primary endpoint was to describe the CASA-Q results in CF adult patients. Secondary endpoints were to evaluate the associations between the CASA-Q and clinical, pulmonary function, exercise tolerance, microbiological and CT-scan features, and also health-related quality of life.

### Statistical analysis

Data were described as numbers (percentages), and mean ± standard deviation. There was not sample size calculation in this pilot study. Differences in clinical characteristics were assessed using chi-square tests or Fisher’s exact tests, as appropriate, for qualitative variables, and Student t-tests or Mann–Whitney U-tests for quantitative variables. Relationships between variables were assessed using the Pearson correlation test. A *p*-value < 0.05 was considered statistically significant. Results were analyzed with SPSSv27.

The Cronbach’s alpha value for CASA-Q and each quality-of-life scores (CFQ-R, SGRQ) was calculated.

A cluster analysis was performed to characterise the clinical phenotype associated with highly impaired CASA-Q. We used the two-step clustering function provided by IBM-SPSS (version 27). This classification method automatically identified subgroups of patients using the scores from the 4 domains of the CASA-Q: cough symptoms, cough impact, sputum symptoms, sputum impact. At the first step, the log-likelihood distance was used to assign participants to the cluster leading to the largest log-likelihood. At the second step, the Bayesian Information Criterion (BIC) was used to assess multiple cluster solutions and automatically determine the optimum number of clusters [16].

## Results

Fifty-one consecutive CF patients were included in the study. Three patients were excluded from the current analysis due to previous lung transplantation (n = 1) or incomplete data (n = 2), 48 patients were analysed. No patient declined to participate in the study.

### Patient characteristics

Demographic, clinical, and functional characteristics are detailed in Table 1. 69% of patients were men. The mean age was 27.8 years with a mean body mass index of 21.8 kg/m^2^. Mean FEV_1_% was 64% of the predicted value. Only two patients (4%) were active smokers. No patient was treated with elexacaftor/tezecaftor/ivacaftor.

**Table 1:** Demographic, clinical and functional characteristics

Variables	n = 48
Male	33 (69)
Age, years	27.8 ± 8.1
BMI, kg/m²	21.8 ± 3.3
Smoker	5 (10)
Active/ Ex smoker	2 (4) / 3 (6)
Pack-years in active/ex-smoker	7 ± 12
CFTR mutation	
ΔF508 homozygous	20 (42)
ΔF508 heterozygous	24 (50)
Other	4 (8)
Pancreatic insufficiency	38 (79)
Diabetes	16 (33)
Osteoporosis	6 (12)
Dyspnea	26 (54)
mMRC ≥ 2	9 (19)
Exacerbation in the last year	33 (69)
Number of episodes per patient	1.5 ± 1.5
FEV_1_, % predicted	64 ± 30
6-minute walking test	
Distance, m	561 ± 88
Distance, % predicted	80

Data are expressed as frequency (percentage) or mean ± standard deviation

BMI: body mass index; m: metres; FEV_1_: forced expiratory volume;

mMRC: modified Medical Research Council

Chronic infections by *S. aureus*, *P. aeruginosa, A. xylosoxidans and S. maltophilia* were present in 74% (n = 37), 40% (n = 20), 4% (n = 2), and 2% (n = 1) of the patients, respectively. Microbiological analysis of sputum at inclusion, available for 42 patients, identified *S. aureus*, *P. aeruginosa* and *A. xylosoxidans* in 76% (n = 32), 40% (n = 17), and 2% (n = 1) respectively. Thoracic CT scans were available for 14 patients. The mean CT-scan score was 25.7 ± 10.6.

### CASA-Q scores and associations with patients’ characteristics

The Cronbach’s alpha value for CASA-Q was 0.966. The mean values for the four domains of the CASA-Q were 58 ± 23 [8–100] for cough symptoms, 77 ± 24 [16–100] for cough impact, 62 ± 25 [8.3–100] for sputum symptoms, and 84 ± 21 [8.3–100] for sputum impact.

The associations between CASA-Q domains, and clinical, functional, microbiological, and CT-scan data are detailed in Table 2. Cough symptoms and impact scores from CASA-Q were significantly more impaired in females (47 ± 22 vs. 63 ± 22, p = 0.029; 65 ± 29 vs. 83 ± 19, p = 0.014; respectively). Analysis of CFTR mutations identified that patients with ΔF508 heterozygous mutation were more impaired on the sputum impact score than homozygous patients (76 ± 27 vs. 93 ± 8, p = 0.008). Each score of the four domains of the CASA-Q was significantly more impaired in patients with a dyspnea score ≥ 2 on mMRC scale (cough symptoms: 39 ± 22 vs. 63 ± 20, p = 0.002; cough impact: 54 ± 27 vs. 84 ± 17, p < 0.001; sputum symptoms: 42 ± 26 vs. 68 ± 21, p = 0.002 and sputum impact: 69 ± 29 vs. 90 ± 12, p = 0.001). Exacerbation in the last year was significantly associated with impairment of cough and sputum symptoms scores (53 ± 23 vs. 68 ± 20, p = 0.03; 56 ± 23 vs. 76 ± 23 respectively, p = 0.01). Distance on the 6-minute walking test was associated with a better score on CASA-Q cough (p = 0.027, r^2^ = 0.461) and sputum impact (p = 0.029, r^2^ = 0.456). To summarize, significantly impaired CASA-Q in CF was associated with female gender, ΔF508 heterozygous mutation, significant dyspnea on exercise, exercise capacity and exacerbation history, but not with lung function and bronchiectasis extend on CT-scan.

**Table 2:** Associations between CASA-Q, and clinical, functional, microbiological and radiological data

	CASA-Q			
	Cough Symptoms	Cough Impact	Sputum Symptoms	Sputum Impact
Gender	**0.029**	**0.014**	0.167	0.061
Age, years	− 0.208; 0.15	− 0.206; 0.159	− 0.224; 0.125	− 0.209; 0.154
BMI, kg/m²	0.123; 0.430	0.025; 0.825	0.088; 0.552	-0.037; 0.803
Smoker	0.649	0.181	0.488	0.076
Ex	0.848	0.894	0.766	0.781
Pack-years	0.312; 0.688	0.044; 0.956	0.525; 0.475	0.171; 0.829
CFTR mutation°	0.197	0.061	0.051	**0.008**
Pancreatic insufficiency	0.644	0.63	0.618	0.229
Diabetes	0.441	0.843	0.517	0.860
Osteoporosis	0.493	0.714	0.785	0.967
Dyspnea (mMRC > 2)	**0.002**	**< 0.001**	**0.002**	**0.001**
Exacerbation in the last year	**0.030**	0,105	**0.010**	0.36
Number of episodes/patient	-0.144; 0.33	− 0.169; 0.252	-0.28; 0.054	-0.183; 0.213
FEV_1_,% predicted	0.205; 0.162	0.001; 0.994	0.154; 0.296	-0.097; 0.511
6-minute walking test^#^				
Distance, m	0.396; 0.061	0.521; **0.011**	0.294; 0.173	0.526; **0.010**
Distance, % predicted	0.31; 0.15	0.461; **0.027**	0.153; 0.486	0.456; **0.029**
Chronic colonisation by SA	0.498	0.349	0.647	0.913
Chronic colonisation by PA	0.358	0.923	0.131	0.784
SA in sputum at inclusion^*^	0.751	0.203	0.356	0.292
PA in sputum at inclusion^*^	0.512	0.316	0.373	0.523
CT-scan score^†^	0.115; 0.695	0.174; 0.551	0.251; 0.387	0.362; 0.204

Data are expressed as “p-value” for qualitative variables and “r²; p-value” for quantitative variables

BMI: body mass index; m: meters; CASA-Q: Cough and sputum assessment questionnaire; FEV_1_: forced expiratory volume; SA: *Staphylococcus aureus*; PA: *Pseudomonas aeruginosa*

° homozygote vs. heterozogote

^#^ data missing for n = 25 patients

^*^ data missing for n = 8 patients

^†^ data missing for n = 14 patients

**Fig. 1:**
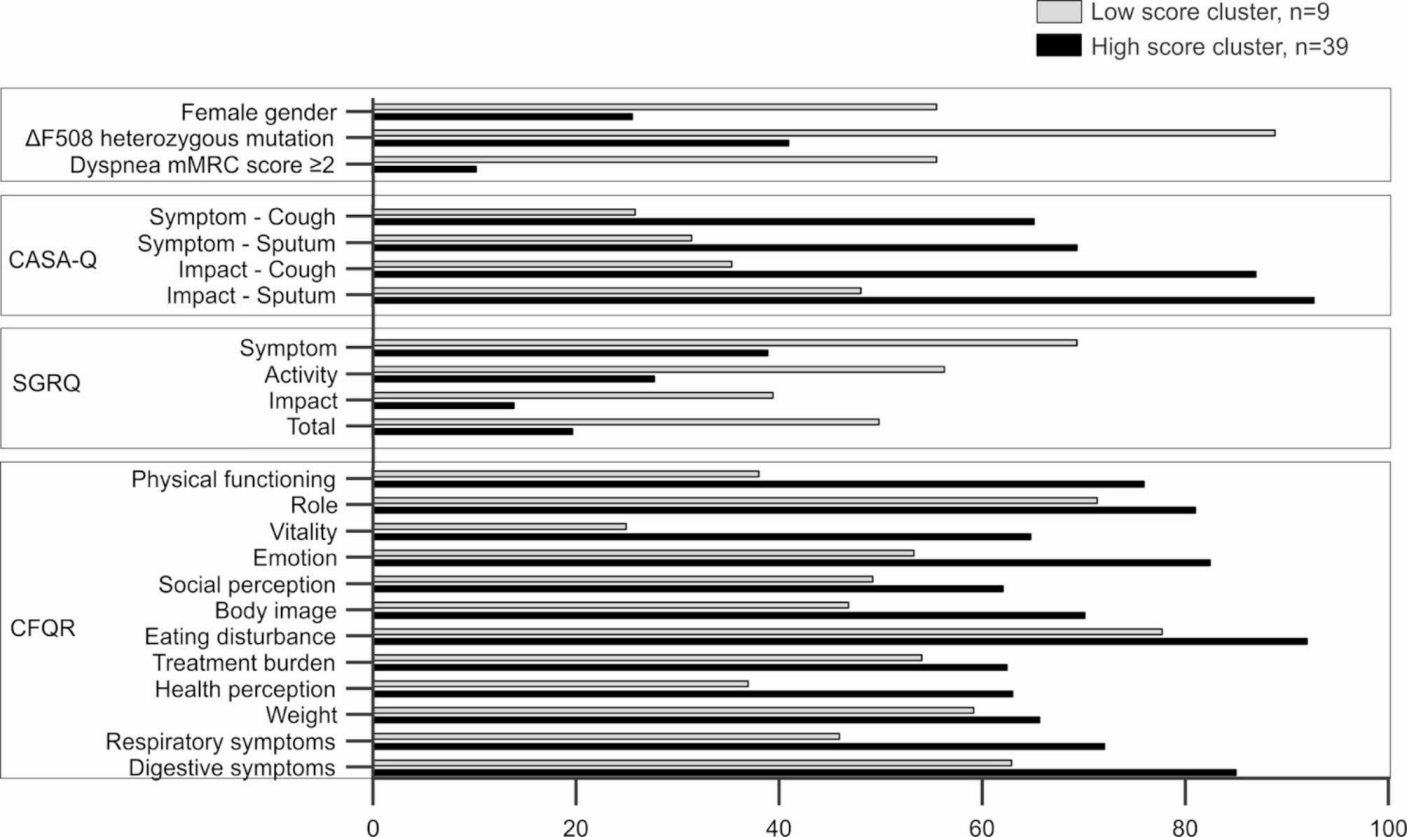
Patient characteristics depending on the CASA-Q score from a cluster analysis. A 2-step clustering of patients was performed characterising 2 clusters: a “low CASA-Q score” cluster (grey bars; n = 9, 18.8%) reflecting the most severely impaired subjects regarding CASA-Q and a “high CASA-Q score” cluster (dark bars; n = 39, 81.2%) reflecting the less severely impaired subjects regarding CASA-Q.

To further characterise the clinical phenotype associated with highly impaired CASA-Q, we undertook a 2-step clustering of patients depending on scores in the 4 domains of the CASA-Q. This analysis resulted in 2 clusters: a “low score” cluster (n = 9, 18.8%) and a “high score” cluster (n = 39, 81.2%) (Fig. 1). The low score cluster (reflecting the most severely impaired subjects regarding CASA-Q) was characterised by more frequent female patients, more frequent ΔF508 heterozygous patients and dyspnea mMRC score ≥ 2.

### Quality of life scales and correlation with CASA-Q

The Cronbach’s alpha value for CFQ-R and SGRQ was 0.602 and 0.911 respectively. Detailed results of quality of life scales are shown in Table S1. On the CFQ-R, “vitality” was the most impaired domain (57 ± 24), and “eating disturbance” was the less impaired domain (89 ± 18). The mean SGRQ score was 25 ± 17 with a predominant impairment in the “symptoms” domain (45 ± 21).

**Fig. 2:**
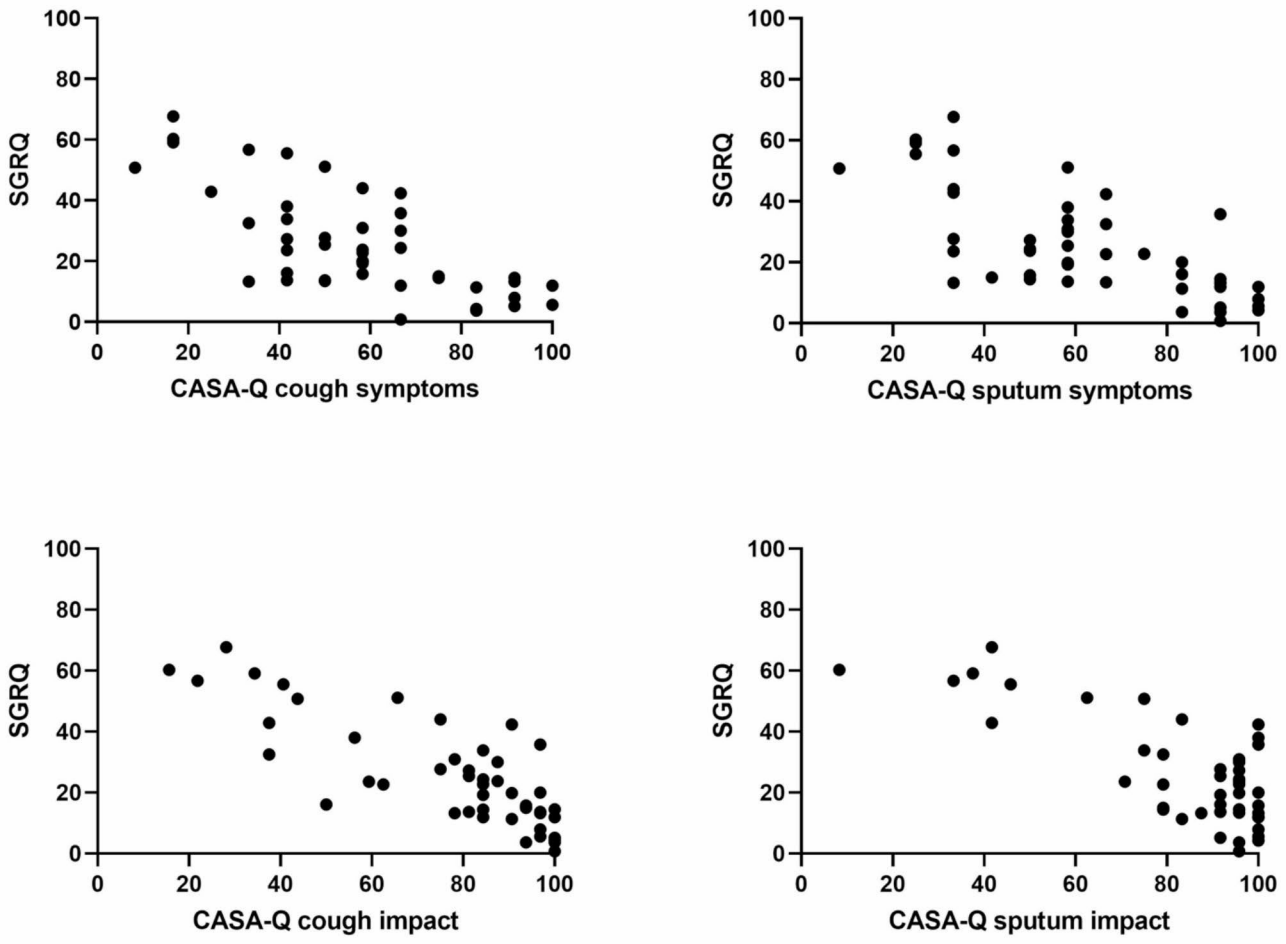
Correlation between CASA-Q and SGRQ total score. Each domain of the CASA-Q (a higher score reflecting a minimal impairment) was close correlated with the total score of SGRQ (a higher score indicating a maximal impairment): cough symptoms: r^2^=-0.736 (p < 0.001); cough impact: r^2^=-0.811 (p < 0.001); sputum symptoms: r^2^=-0.713 (p < 0.001); sputum impact: r^2^=-0.755 (p < 0.001)

The correlation between the CASA-Q and the quality of life scales CFQ-R and SGRQ are detailed in Table 3. The total score and all domains of the SGRQ correlated with each domain of the CASA-Q (p < 0.001) suggesting a close association between the 2 scores (Fig. 2). Except for “role perception”, “treatment burden” and “weight”, domains of CFQ-R significantly correlated with all or many domains of CASA-Q score.

**Table 3:** Association between CASA-Q and quality of life scales

	CASA-Q			
	Cough symptoms	Cough Impact	Sputum symptoms	Sputum Impact
SGRQ				
Total	-0.736; **< 0.001**	-0.811; **< 0.001**	-0.713; **< 0.001**	-0.755; **< 0.001**
Impact	-0.610; **< 0.001**	-0.764; **< 0.001**	-0.547; **< 0.001**	-0.766; **< 0.001**
Activities	-0.558; **< 0.001**	-0.611; **< 0.001**	-0.474; **0.001**	-0.574 ; **< 0.001**
Symptoms	-0.744; **< 0.001**	-0.714; **< 0.001**	-0.831; **< 0.001**	-0.648; **< 0.001**
CFQ-R				
Physical functioning	0.605; **< 0.001**	0.662; **< 0.001**	0.533; **< 0.001**	0.554; **< 0.001**
Role perception	-0.041; 0.8	0.181; 0.263	-0.017; 0.917	0.129; 0.428
Vitality	0.605; **< 0.001**	0.759; **< 0.001**	0.640; **< 0.001**	0.648; **< 0.001**
Emotion	0.416; **0.004**	0.544; **< 0.001**	0.464; **< 0.001**	0.547; **< 0.001**
Social perception	0.371; **0.011**	0.461; **0.001**	0.361; **0.014**	0.409; **0.005**
Body image	0.387; **0.007**	0.468; **0.001**	0.316; **0.03**	0.370; **0.01**
Eating disturbance	0.326; **0.033**	0.275; 0.074	0.202; 0.194	0.416; **0.006**
Treatment burden	0.139; 0.373	0.198; 0.203	0.214; 0.168	0.189; 0.225
Health perception	0.546; **< 0.001**	0.598; **< 0.001**	0.543; **< 0.001**	0.502; **<0.001**
Weight	0.091; 0.546	− 0.007; 0.964	0.069; 0.49	0.004; 0.772
Respiratory symptoms	0.732; **< 0.001**	0.725; **< 0.001**	0.825; **< 0.001**	0.682; **< 0.001**
Digestive symptoms	0.255; 0.095	0.493; **0.001**	0.313; **0.038**	0.436; **0.03**

Data are expressed as “r²; p-value”

CASA-Q: Cough and sputum assessment questionnaire; CFQ-R: Cystic fibrosis questionnaire revised; SGRQ: Saint George Respiratory Questionnaire;

The cluster analysis revealed that the patients in the low score cluster had a global impairment in all domains of the SGRQ and CFQ-R (Fig. 1).

## Discussion

This pilot study analyses the CASA-Q results in an adult CF population. Our results demonstrate that CASA-Q is highly correlated with respiratory symptoms including dyspnea and exacerbations, and also with the quality of life scores. Interestingly, we identified a clinical phenotype associated with an impairment of CASA-Q score and quality of life: female gender, ΔF508 heterozygous mutation and more dyspnea assessed by the mMRC scale.

Cough and sputum are frequently encountered symptoms in CF patients [2, 17]. Cough was reported as the highest symptom burden in adults [18]. In clinical trials, assessment of treatment effectiveness is mainly based on the improvement of lung function and weight gain. Measurement of cough is usually not based on a specific score but is included in daily respiratory symptoms questionnaire [19] or in global quality of life scales [20]. Whereas the CFQ-R and SGRQ scores focus on overall health-related quality of life, the CASA-Q allows a specific evaluation of cough, sputum symptoms and impact. In CF, CASA-Q was used in one clinical study to compare two techniques of daily airway clearance [10]. Our study showed that CASA-Q is correlated with respiratory clinical characteristics such as dyspnea on mMRC scale ≥ 2 (on the four domains) and occurrence of exacerbation in the last year (on both “symptoms” domains). In clinical practice, this specific questionnaire may be useful as part of routine screening at stable state in CF patients or as an objective measurement of short-term cough improvement with new therapies [21]. The use of CASA-Q during and after an exacerbation as shown in COPD [14] remained be assessed.

Interestingly, we observed that the impact of cough was greater in females. Previous study showed that females were more embarrassed about coughing than males and suppressed coughing in public situations to avoid negative attention. This behavior was associated with reduced health-related quality of life [22]. Moreover prevalence of urinary incontinence often related to coughing is greater in adult females than males in CF [23].

We showed no correlation between CASA-Q and bacteriological sputum data. Of note, chronic pulmonary colonization with *P. aeruginosa* is associated with pulmonary deterioration and poor clinical prognosis [24]. The relationship between CASA-Q and functional data was divergent. We showed no correlation between CASA-Q and FEV_1_%. By contrast, “impact” domains of CASA-Q were correlated with a better distance on the 6-minute walking test. To our knowledge, no reported study assessed the impact of cough and sputum on a 6-minute walking test in CF. Additional studies including a larger group of patients would be interesting to draw a definitive conclusion regarding the relationship between the CASA-Q questionnaire results and FEV_1_ or the 6MWT, and would allow to determine a potential ceiling effect.

Interestingly, we showed a close correlation between CASA-Q and two of the main quality of life scales in CF adult patients [20]. The CASA-Q strongly correlated with SGRQ total score and all domains, suggesting a high impact of cough and sputum in all dimensions of the SGRQ. It could be argued that the relationship between the CASA-Q scores and the SGRQ scores was due to similar questions on cough and sputum. However, only two questions in the SGRQ explore cough and sputum symptoms, with a minimal effect on the total score. In COPD, each CASA-Q domain significantly correlated with SGRQ except for CASA-Q symptoms and SGRQ activities [6]. Of note, only the cough impact domain was independently associated with the total SGRQ score in COPD [25]. We also found a good correlation between CASA-Q and most of the CFQ-R domains. Some subsections of the CFQ-R were not or only weakly correlated with the CASA-Q scores (role, treatment burden, digestive symptoms, eating, and weight). It may be explained by the fact that CFQ-R includes some extra-respiratory dimensions such as digestive symptoms. Moreover, a moderate association was previously described between the “weight” domain on CFQ-R and BMI [26].

There are some limitations to our study. First, this pilot cross-sectional study was conducted in a single centre with a relatively small sample size. A larger multicentric study should be conducted to confirm these preliminary results. Second, we did not compare the CASA-Q with the Leicester Cough Questionnaire, validated for CF patients after the onset of our study [27]. In San Miguel-Pagola et al. study, the CASA-Q seemed to be more appropriate to detect the short-term effect of clearance sessions than the Leicester Cough Questionnaire [10]. Third, no association was found between CASA-Q and bronchiectasis CT scan score, but the small sample size of 14 patients with available CT-scans does not allow to draw a definitive conclusion. Fourth, our study was conducted before the large use of CFTR modulator therapies including the combination of elexacaftor/tezecaftor/ivacaftor. It will be interesting to see how these specific drugs impact cough and sputum based on the CASA-Q. Lastly, the minimal clinical important difference (MCID) has not been established for the CASA-Q in CF. In COPD patients following pulmonary rehabilitation, MCID was interpreted as relevant from 10.6, 10.1, 9.5, and 7.8 for cough symptoms, cough impact, sputum symptoms, and sputum impact respectively [28], but additional studies are needed to determine MCID of the CASA-Q in CF.

## Conclusions

This pilot study supports the use of CASA-Q for cough and sputum assessment in CF adult patients in a stable state. We showed that impaired CASA-Q scores reflected more breathlessness and past exacerbations. CASA-Q was strongly correlated with quality of life scales both specific to CF disease (CFQ-R) or related to respiratory disease (SGRQ). Additional larger studies are needed to validate these results. It would be also interesting to assess the CASA-Q during and after a pulmonary exacerbation, and before and after initiating CF therapeutic strategies.

### Electronic supplementary material

Below is the link to the electronic supplementary material.

Supplementary Material 1

## Data Availability

The datasets used and/or analysed during the current study are available from the corresponding author on reasonable request.

## Declarations

### Patient agreement

Each patient signed a written informed consent.

## List of abbreviations

BMI: Body mass index
CASA-Q: Cough and sputum assessment questionnaire
CF: Cystic fibrosis
CT scan: Computerized tomography scan
CFQ-R: Cystic fibrosis questionnaire revised
FEV_1_: Forced expiratory volume at first second
QOL: Quality of life
PRO: Patient-related outcome
SGRQ: Saint George Questionnaire Respiratory Questionnaire
