# Supplementary material for: Current cough and sputum assessed by the cough and sputum assessment-questionnaire (CASA-Q) is associated with quality of life impairment in cystic fibrosis
Source: BMC Pulm Med. 2023 Nov 21;23:457. doi: 10.1186/s12890-023-02701-3 (PMC10664648; doi:10.1186/s12890-023-02701-3)
Supplement: Supplementary file 1 — Supplementary Material 1 [file 12890_2023_2701_MOESM1_ESM.docx]

**Table S1- CFQ-R and SGRQ scales**

| **Variables** |  | |  | |
| --- | --- | --- | --- | --- |
| CFQ-R |  | |  | |
| Physical functioning | | 70 ± 26 | |  |
| Role perception | | 79 ± 21 | |  |
| Vitality | | 57 ± 24 | |  |
| Emotion | | 77 ± 23 | |  |
| Social Perception | | 60 ± 12 | |  |
| Body image | | 66 ± 25 | |  |
| Eating disturbance | | 90 ± 18 | |  |
| Treatment burden | | 61 ± 18 | |  |
| Health perception | | 58 ± 25 | |  |
| Weight | | 64 ± 37 | |  |
| Respiratory Symptoms | | 68 ± 15 | |  |
| Digestive Symptoms | | 80 ± 20 | |  |
| SGRQ | |  | |  |
| Total | | 25 ± 17 | |  |
| Impact | | 20 ± 16 | |  |
| Activity | | 34 ± 21 | |  |
| Symptoms | | 45 ± 21 | |  |
| Data are expressed as mean ± standard deviation.  CFQ-R: Cystic fibrosis Questionnaire revised; SGRQ: Saint George Respiratory Questionnaire; | | | | |
